# Influence of Retinal Image Shifts and Extra-Retinal Eye Movement Signals on Binocular Rivalry Alternations

**DOI:** 10.1371/journal.pone.0061702

**Published:** 2013-04-12

**Authors:** Joke P. Kalisvaart, Jeroen Goossens

**Affiliations:** Radboud University Nijmegen Medical Centre, Donders Institute for Brain, Cognition and Behaviour, Dept. Cognitive Neuroscience, section Biophysics, Nijmegen, The Netherlands; Macquarie University, Australia

## Abstract

Previous studies have indicated that saccadic eye movements correlate positively with perceptual alternations in binocular rivalry, presumably because the foveal image changes resulting from saccades, rather than the eye movement themselves, cause switches in awareness. Recently, however, we found evidence that retinal image shifts elicit so-called onset rivalry and not percept switches as such. These findings raise the interesting question whether onset rivalry may account for correlations between saccades and percept switches.

We therefore studied binocular rivalry when subjects made eye movements across a visual stimulus and compared it with the rivalry in a ‘replay’ condition in which subjects maintained fixation while the same retinal displacements were reproduced by stimulus displacements on the screen. We used dichoptic random-dot motion stimuli viewed through a stereoscope, and measured eye and eyelid movements with scleral search-coils.

Positive correlations between retinal image shifts and perceptual switches were observed for both saccades and stimulus jumps, but only for switches towards the subjects' preferred eye at stimulus onset. A similar asymmetry was observed for blink-induced stimulus interruptions. Moreover, for saccades, amplitude appeared crucial as the positive correlation persisted for small stimulus jumps, but not for small saccades (amplitudes < 1°). These findings corroborate our tenet that saccades elicit a form of onset rivalry, and that rivalry is modulated by extra-retinal eye movement signals.

## Introduction

When the left and the right eye are presented with different images that cannot be fused into a single three-dimensional scene, binocular rivalry can arise: the images are not merged into a single percept, but instead seen alternately. This phenomenon is studied extensively because it can dissociate the visual input from the perceptual output, which might give us insight in the origin of visual awareness. Thus far, however, the exact mechanisms underlying binocular rivalry are not fully understood. Models of binocular rivalry typically assume that rivalry arises from competition between retinotopically-organised cell populations [1], [2], [3]. In these models, mutual inhibition between cell-populations that code for the different percepts prevent simultaneous perception of both stimuli, while self-adaptation of the active cell-population causes the dominant percept to fade after a certain period and to be replaced by the other percept (but see also [4], for a different perspective). However, none of these models consider the effect of saccades.

Although there is convincing evidence that perceptual alternations can occur without eye movements [5], [6], [7], several studies have reported correlations between saccades and perceptual switches (e.g., [8], [9]). Van Dam and van Ee [10] found a marked increase in saccade occurrence just before subjects reported a perceptual switch in binocular rivalry conditions, suggesting that saccadic eye movements cause perceptual switches. A later study indicated, however, that a saccade only causes a perceptual switch if the eye movement leads to a retinal image change on the fovea [11]. Indeed, a saccade moves the stimulus across the retina in such a way that after the eye movement different retinotopic groups of cells will be stimulated. These neurons will have a different visual history and will therefore be in a different adaptation state. Adaptation studies indicate that, at least for lower-order stimuli such as the gratings applied by van Dam and van Ee [11], adaption only occurs at retinotopically matched locations [12], [13] and rivalry has been found to slow down if the stimulus is moving, preventing adaptation [14].

A series of recent studies [15], [16], [17], [18] have shown, however, that rivalry during sustained viewing and rivalry at stimulus onset are at least partly different. For example, Mamassian and Goutcher [15] found that contrast differences between the two stimuli cause a strong eye bias at stimulus onset that wears off during the course of the trial toward a more equal dominance of the two eyes. In addition, we have recently shown that retinal image shifts produced by a saccade elicit a form of onset rivalry, rather than percept switches per se [19]; when subjects made a 4° saccade after prolonged viewing of a rivalrous stimulus, eye dominance after the saccade was biased in the same subject-specific direction as the eye dominance at stimulus onset. These findings raise the interesting question whether there is a relation between onset rivalry and the previously reported positive correlations between saccades and perceptual switches in binocular rivalry.

In the present study we therefore investigate the consequences of multiple saccades made during sustained viewing. We asked subjects to make saccades within a binocular rivalry stimulus and we studied the timing of the saccades in relation to perceptual switches. We compared active and passive retinal image shifts (together also called *shifts* throughout this article). Active shifts were caused by saccades, passive shifts by moving the stimulus across the screen in a saccade like fashion (‘replay’ condition). The notion that saccades may elicit onset rivalry rather than percept switches per se, predicts that saccades will occur more frequently before switches towards the subject's preferred eye than before switches to the non-preferred eye. Moreover, if the positive correlations between saccades and perceptual switches arise solely from the consequences of retinal image shifts, the effects in saccade and replay conditions should be the same.

In previous studies, using intermittent stimulus presentations, a short (<0.5 s) stimulus interruption strongly increased the probability of percept alternations [18], [20]. We therefore also studied the effect of eye blinks. Blinks cause a short interruption of the stimulus on the retina but, unlike saccades, do not move the stimulus to a different location on the retina [21], [22].

We report significant correlations between retinal image shifts and perceptual switches for saccades and stimulus jumps, but positive correlations were only observed for switches towards the subjects' preferred eye at stimulus onset. A similar asymmetry was observed for blinks. Our findings thus support the conclusion that retinal image shifts and brief image blanking elicit onset rivalry. We also observed a remarkable difference between small versus large image shifts. For large shifts (>1°), we found a comparable increase in the probability of saccades and stimulus jumps just before a perceptual switch. This increase was also present for small (<1°) stimulus jumps, but virtually absent for small saccades. The latter results further support the notion that extra-retinal eye movement signals are involved in binocular (onset) rivalry.

## Methods

### Subjects and ethics statement

Four adult human subjects with normal or corrected to normal visual acuity participated in the experiments. All subjects were informed about the experimental procedures and gave written informed consent before the experiments. Procedures were approved by the Radboud University Medical Centre.

### Setup

Subjects were seated in a dark room at 52 cm from a projection screen on which visual stimuli were back projected with an LCD projector (Panasonic PT-AX100E). The subject watched the screen through a front-mirror stereoscope (HyperView, Berezin, U.S.). The head rested on a chin support to restrict head movements.

Eye movements were recorded with the scleral search coil technique [23]. Coils were inserted after one drop of topical anesthetic (Oxybuprocaine hydrochloride 0.4%, Thea, Belgium). Once the coil was in place, a drop of artificial tear (Methylcellulose 0.5%, Thea, Belgium) was applied to minimize ocular discomfort and to avoid reduction of visual acuity. To record blinks, a tiny coil (3 mm diameter) was attached to the upper eyelid with a small piece of skin tape (Leukopor, Beiersdorff AG). Eye and eyelid position signals were low-pass filtered, amplified, and sampled at 500 Hz per channel using a CED-1401 data acquisition system. The resolution of the horizontal and vertical eye position signals was better than 0.3 minutes of arc (root mean square measure).

### Stimuli

**Figure 1:**
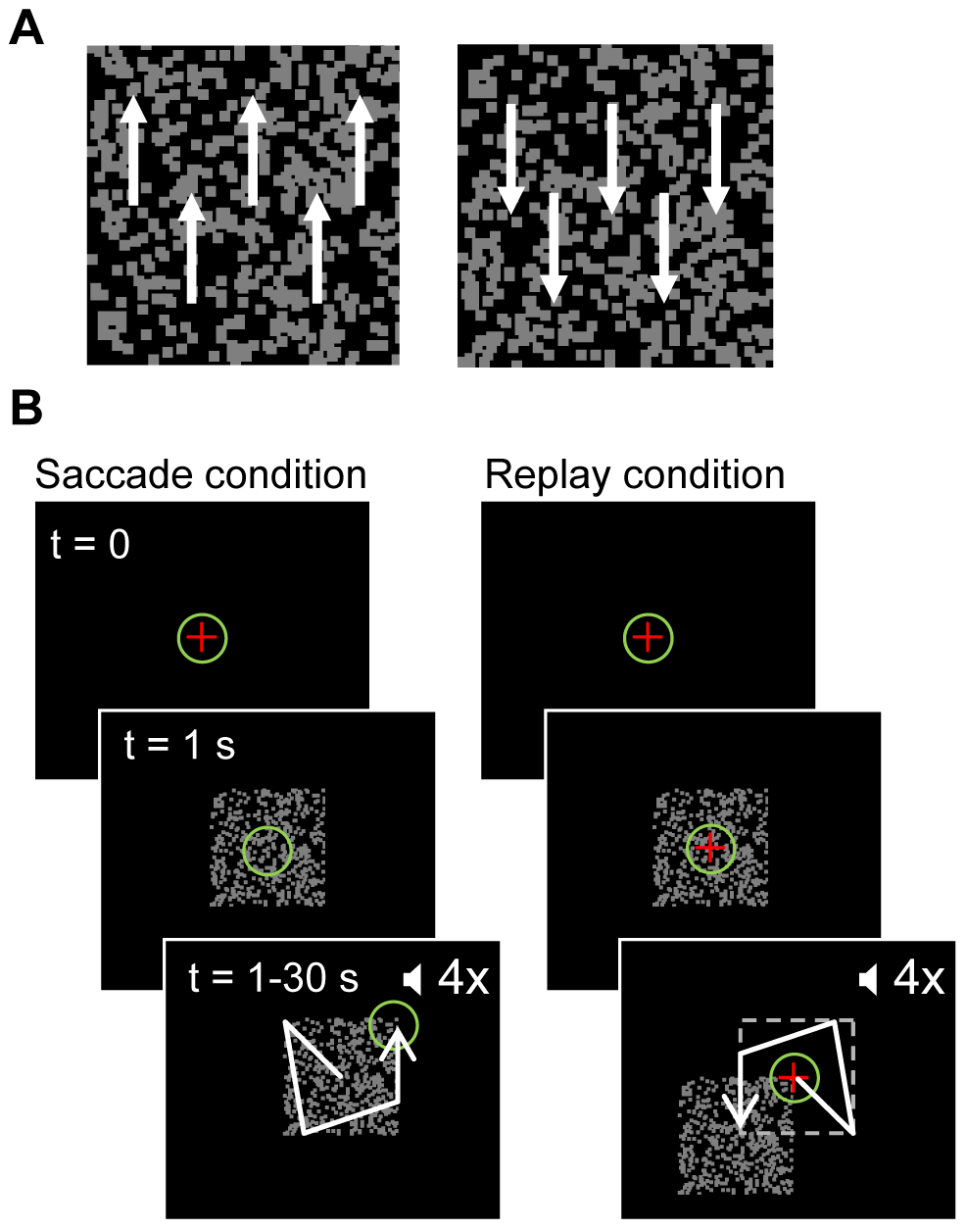
Stimulus and paradigm. A: Example of the motion stimulus used in this study. The arrows indicating motion direction in the left and right eye were not present in the real stimulus. B: Time course of a saccade trial and a replay trial. Each trial starts with the presentation of a central fixation cross. After 1 second, the dichoptic stimulus appears, and the subject starts judging motion direction. During the rest of the 30 s trial, either the gaze or the stimulus moves while the subject keeps indicating the perceived motion direction. Four auditory cues per trial were used in the saccade trials to indicate saccade moment. These cues were also presented in the stimulus jump trials, where they served as a warning for upcoming large stimulus jumps. + fixation point, O subject's gaze, 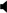 auditory cue. White arrow: illustration of possible gaze path in saccade trials and corresponding stimulus path in replay trials.

The dichoptic stimuli consisted of 4×4° squares filled with 500 random dots moving coherently in opposite directions against a black background (Figure 1A). They were generated with Matlab (The MathWorks) using the Psychophysics Toolbox extensions [24], [25]. The dots were 0.14° white squares moving vertically with a speed of 2.75° per second (1 pixel/frame) and had asynchronous lifetimes of 0.33 s. Motion direction was pseudo-randomly alternated between the eyes from trial to trial. Screen refresh rate was 60 Hz. Stimulus contrast was the same for images presented to the left and the right eye (luminance of dots and background were 98 cd/m^2^ and 1.3 cd/m^2^, respectively; Minolta LS-100 Luminance meter). We used dense random-dot motion stimuli, rather than e.g. (moving) gratings or face/house stimuli, because for these stimuli the foveal motion signal is hardly altered by eye movements within the aperture.

### Task

Subjects continuously indicated their dominant percept by pressing one of two mouse buttons while watching the stimulus. Button presses were recorded by the stimulus program. Subjects were instructed to indicate the most dominant percept if the suppression was not complete. Piecemeal percepts – if present – occurred mainly during the brief perceptual transitions. There were two different conditions, a saccade condition and a ‘replay’ condition, illustrated in Figure 1B. In each condition, trials lasted 30 seconds.

In the *saccade condition*, the subject was instructed to make a few large saccades to random location within the stimulus. From pilot experiments, it appeared that subjects found it very hard to simultaneously indicate their percept and plan saccades independent of their percept alternations. Saccades were often postponed to just after a button press, when the rivaling percept was most stable. To avoid this biased timing of saccades, and to ease the task for the subject, we provided auditory saccade cues (1 kHz tone lasting 0.25 s) at four pseudo-random moments during each trial. Subjects were instructed to make a saccade immediately after the cue and then maintain fixation at that location until the next cue. The central fixation cross was only present at the beginning of saccade trials.

In the *replay condition*, subjects were instructed to fixate the central fixation cross that remained visible throughout these trials, while the stimulus jumped around in a way that resembled the eye movements in the saccade trials. The sequence of stimulus jumps was programmed according to the eye displacements recorded in a previous saccade trial, including small saccades that subjects inadvertently made, but excluding slow-velocity eye drifts. Auditory cues were replayed as well, providing the subjects with a warning cue for upcoming large stimulus jumps.

Saccade trials and replay trials were alternated within blocks of 8 trials. Each subject completed a minimum of 160 trials, across several sessions.

### Control experiments

Prior to the main experiment, we measured the dominance duration distributions of our subjects under static viewing conditions. In these control experiments, subjects were required to fixate a fixation cross either at the centre or on the edge of the stimulus for the duration of the trial.

We also measured the subjects' reaction times to physical flips in the direction of motion. In this experiment, the dot patterns in both eyes moved in the same direction, and subjects kept fixating the central fixation cross. The motion direction was changed at random moments in time and the subjects indicated their percept in the same way as they did during the binocular rivalry trials. The reaction time obtained from this experiment served as an estimate of how long before a button press the perceptual switch occurred.

### Data analysis

Saccades were detected offline on the basis of calibrated eye position signals with custom software. Detection of saccade onsets and offsets was based on velocity and acceleration criteria. All saccade markers were examined by the experimenters and, if necessary, corrected. Saccades smaller than 0.2° were considered micro-saccades and were excluded from the analysis. Eye movements caused by blinks could be readily dissociated from saccade-related movements of the eyes based on their double-peaked velocity profile [21] and were removed manually. Blinks were detected separately, based on the amplitude of the eyelid signal. Further analysis was done with Matlab using custom software.

**Figure 2:**
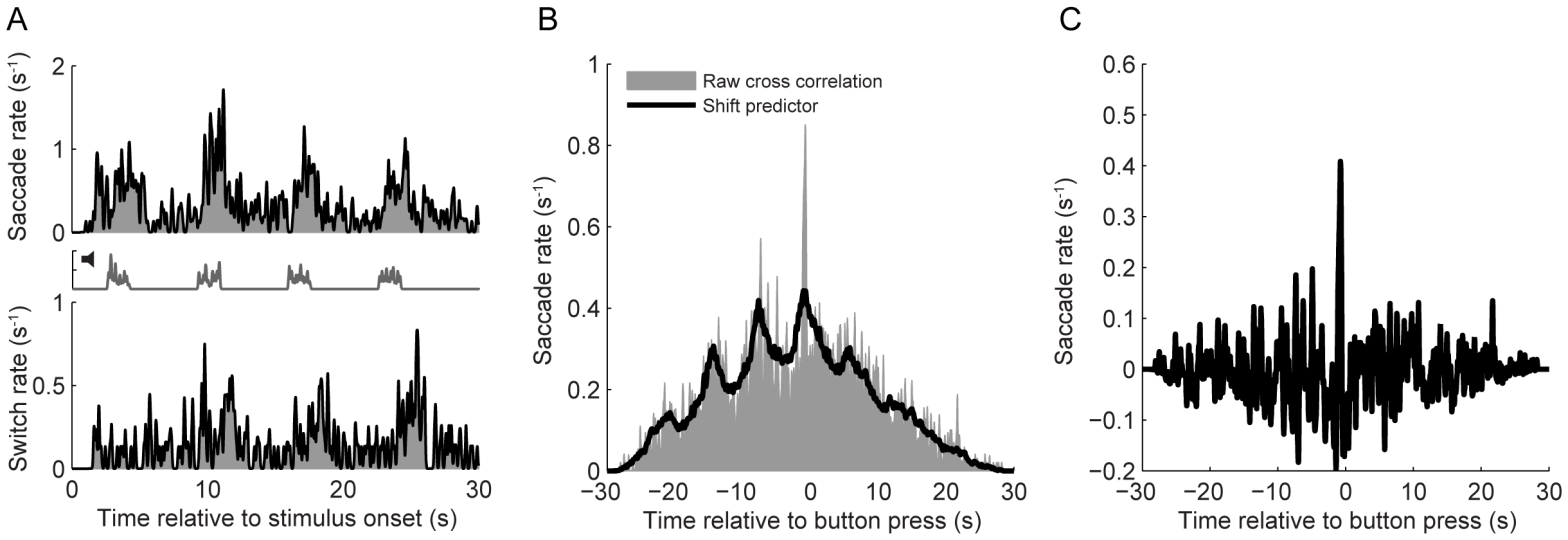
Illustration of the applied cross correlation analysis. A: Peristimulus time histograms (PSTH) showing the average saccade (top) and switch (bottom) rates as a function of time relative to stimulus onset together with PSTHs of the auditory cues (center, gray line). B: Raw cross correlation between saccades and perceptual switches (gray) together with the shift-predictor (black). Data were normalized according to the total number of percept switches such that the vertical axis represents the conditional saccade rate (in saccades per second) as a function of time relative to the button press. C: Covariogram. Corrected cross-correlation histogram obtained by subtracting the shift-predictor from the raw cross correlogram. Data from subject DB. Large saccades (>1°), percept switches from left to right eye.

To examine the relation between saccades/stimulus jumps and percept switches, cross-correlation histograms were made in which the occurrences of saccades and stimulus jumps were plotted relative to the moments of a button press. It is important to realize, however, that the saccade and switch rates need not be constant over time (Figure 2A), and that saccade events and percept switches are both, in a way, driven by the stimulus. In principle, this co-stimulation of the visual system and the saccadic system could cause a peak in the correlation histogram all by itself; the common input might introduce a relation between saccades and percept switches even if no physical relation exists.

To account for this potential pitfall, we applied cross correlation methods that are often used in the analysis of pairs of neuronal firing [26], [27]. In short, we first computed the raw cross correlogram and subtracted from this the so-called shift predictor. This procedure is illustrated in Figure 2B and 2C. The raw cross-correlogram was obtained by cross-correlating the sequence of percept switches in one trial with the sequence of saccades occurrences in that *same* trial, and averaging the results across all trials. The shift predictor, on the other hand, was obtained by cross-correlating the sequence of percept switches in one trial with the sequence of saccades occurring in a *different* trial (which destroys the physiological relation between the two events), and averaging the results across all possible trial combinations. The shift predictor thus predicts the shape of the cross correlation histogram given the null-hypothesis that there is no physical relationship between the two different events [27], [28]. For computational efficiency, this calculation of the shift predictor was done by taking the cross product of the saccade and switch peristimulus time histogram, which yields the same result. All histograms were smoothed with a Gaussian smoothing kernel (width σ = 0.05s). Previous studies have based their analyses on raw cross correlograms (cf. Figure 2B), and have applied a somewhat arbitrary normalization of these uncorrected correlograms (e.g. [9], [10]). Here we ensure that the resulting covariograms reflect the conditional saccade rate above or below that predicted in the absence of any relation. This method also allowed us to directly compare the size of the effects found in different conditions. To test whether covariations were statistically significant, we applied a bootstrap excursion test (BE-test for short) as described by Ventura et al. [29]. Differences between covariograms were also evaluated with this test.

To further address the question how passive versus active retinal image shifts affected the durations of dominance states, we also calculated the mean dominance duration of the left and right eye percepts for each trial. In addition, we quantified the mean delay between retinal image shifts and the first ensuing percept switch. We dubbed this variable mean dominance survival time since it indicates how long the current percept survives after saccades or stimulus jumps.

Mean dominance durations and mean survival times from each trial were then sorted according to conditions, and averaged per condition. Standard errors for these measures were computed from the variance of the mean values across trials. Mean dominance durations in the different conditions were compared using Student's t-test. Two-way ANOVAs were used to test for differences between the mean survival times in the saccade and the replay condition. Independent variables in the ANOVA analysis were condition and subject.

## Results

**Figure 3:**
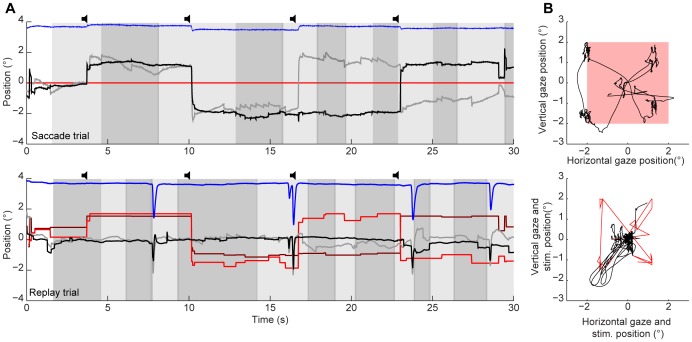
Percept alternations, together with eye and eyelid movements in a saccade and replay trial. A: Horizontal (black) and vertical (gray) eye position, horizontal (dark red) and vertical (bright red) stimulus positions, and vertical eyelid position (blue, in arbitrary units), during the course of a saccade trial (top) and a [3]replay trial (bottom). Light and dark gray areas indicate left and right eye dominance epochs, respectively. 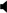 auditory cue. B: Two dimensional eye (black) and stimulus (red) position relative to the centre of the screen in the same saccade (top) and replay trials (bottom) as shown in A. Data from subject SR.

Figure 3 illustrates the time course (Figure 3A) and the 2D-trajectories (Figure 3B) of the eye and stimulus displacements as well as the percept alternations during a saccade (top) and replay (bottom) trial. Apart from the four saccades that we asked for during each saccade trial (by means of the auditory cues), subjects inadvertently made many extra saccades, almost always relatively small ones.

**Figure 4:**
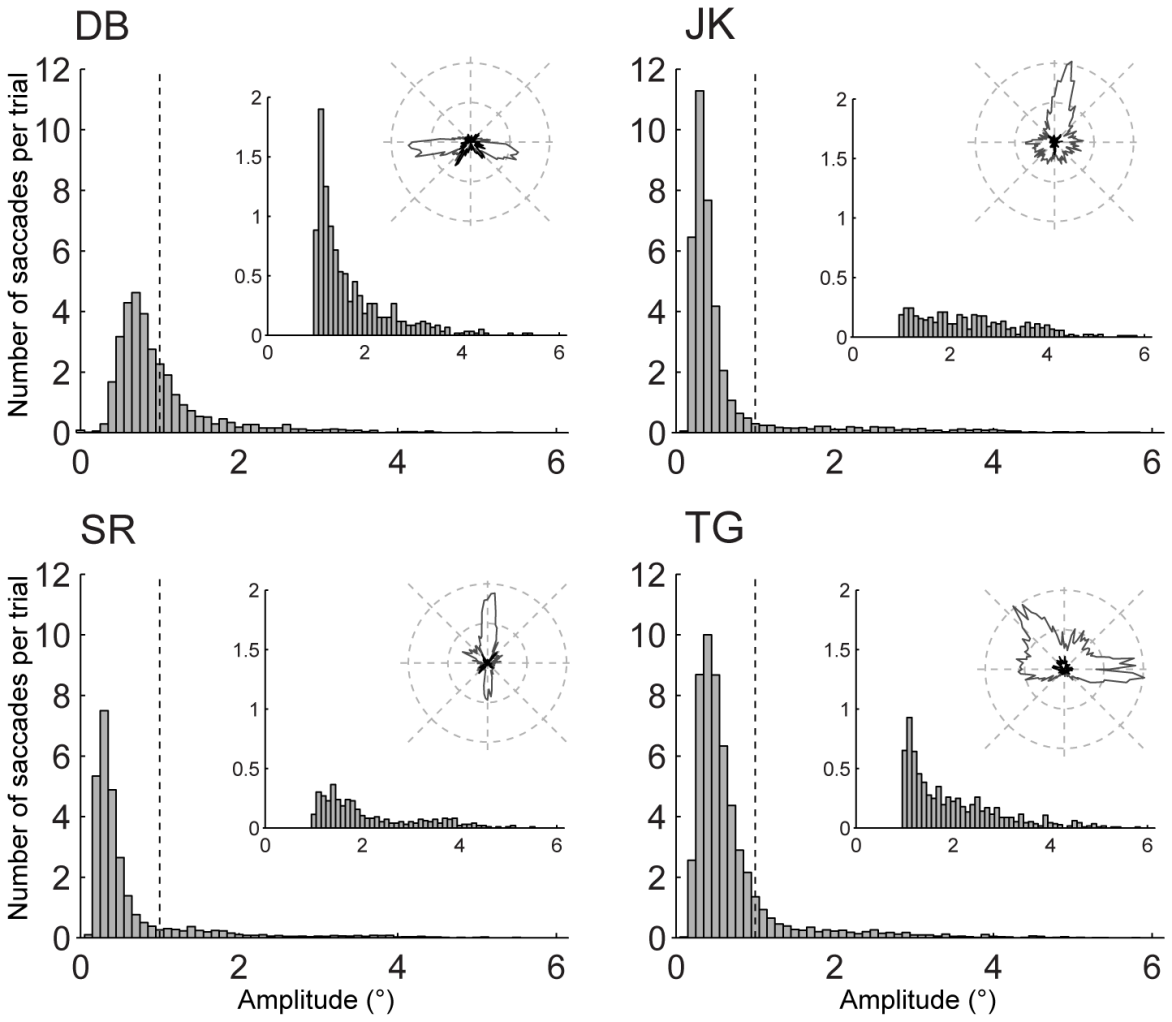
Distribution of saccade amplitudes in saccade trials for each subject. Bin size 0.1°. Insets show the distribution of saccades >1° on a different scale and polar histograms of saccade direction for small (gray) and large (black) saccades.

Figure 4 shows the distribution of saccade amplitude and direction in the saccade condition for each subject. Note that the amplitude distributions were highly skewed, with most saccades being <1°. Saccade directions were also not uniformly distributed, but there was no systematic bias towards the up/down directions of the motion stimuli (except for subject SR). Because the stimulus jumps in replay trials were programmed after the eye movements in saccade trials, the same distribution of stimulus jumps resulted. In our analysis, we divided the retinal image shifts produced by saccades and stimulus jumps into two groups: small shifts with amplitudes less than 1°, and large shifts with amplitudes equal to or larger than 1°. We decided on a 1° amplitude threshold because the amplitude distributions contained a sharp peak at amplitudes <1° which was followed by a long, more or less flat tail starting at an amplitude of about 1°. The exact boundary value that we used to discriminate between large and small saccades was, within limits of about 0.5–1.5°, not crucial for the results presented below.

From analyzing the first button presses at the start of each trial, we inferred that subjects had an eye preference bias at stimulus onset. Subjects DB, JK and SR had right eye preferences at stimulus onset of 77%, 71% and 56%, respectively, whereas TG had a left eye onset preference of 58%. These onset biases disappeared quickly during the course of the trial, resulting in a much more balanced dominance of the two eyes during sustained rivalry. To account for these eye biases, we analyzed perceptual switches from the preferred to the non-preferred eye and perceptual switches from the non-preferred to the preferred eye separately.

### Temporal cross-correlation

**Figure 5:**
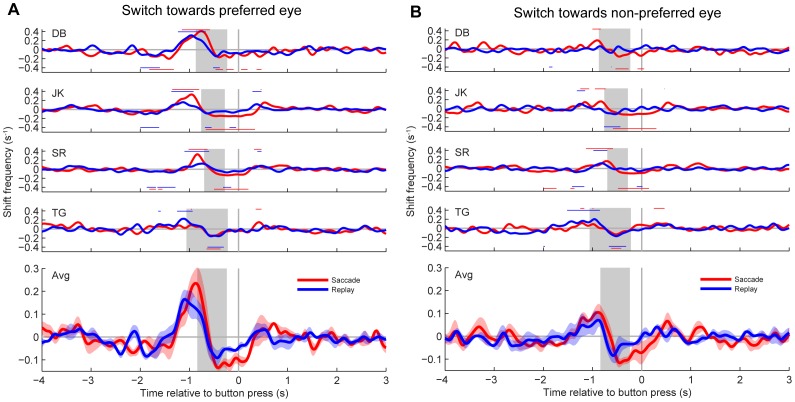
Covariograms of percept switches and large (>1°) retinal image shifts. Shifts were produced by saccades (red) or stimulus jumps (blue). A: Switches from the non-preferred to the preferred eye. B: Switches from the preferred to the non-preferred eye. Top panels show the results from individual subjects. Bottom panels plot the mean across all four subjects. Shaded areas denote ± 1 SEM. Gray horizontal lines represent zero deviation from the baseline shift predictor (c.f., Methods, Figure 2). The vertical gray bar is an estimate (mean±SD) of the moment that the actual percept switch occurred relative to the moment of the button press (i.e., the reaction time obtained from the stimulus flip condition in control experiments). Red and blue horizontal lines above the graphs from individual subjects indicate a significant peak in that time window for saccade or stimulus jump frequency, respectively; similarly, red and blue lines underneath the graphs indicate significant troughs. (BE-test, p<0.05).

**Figure 6:**
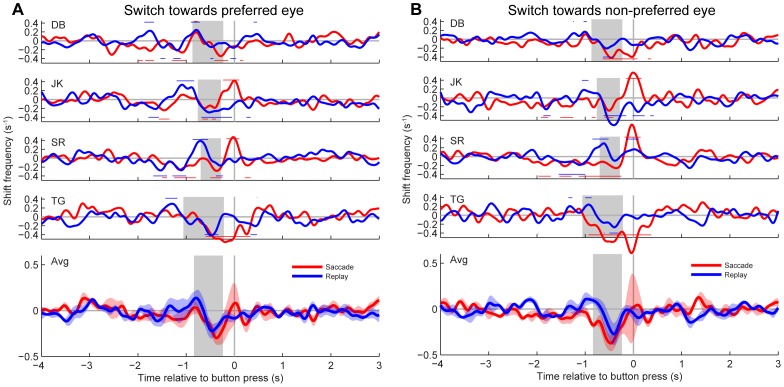
Covariograms of percept switches and small (<1°) retinal image shifts. Shifts were produced by saccades (red) or stimulus jumps (blue). A: Switches toward the preferred eye. B: Switches towards the non-preferred eye. Same lay-out as Figure 5, but note the different scaling of the vertical axes.

Figures 5 and 6 show smoothed covariograms (Methods) obtained for all four subjects as well as the mean across subjects for the time interval −4 to +3 seconds relative to the button press. Red curves show data from saccade trials. Blue curves show data from replay trials. Epochs with statistically significant increases from the shift-predictor baseline (Methods; BE-test, p<0.05) are indicated with horizontal red and blue lines above the covariograms for the saccade and replay condition, respectively. Horizontal lines underneath the covariograms indicate significant decreases from the baseline. The vertical gray bar indicates the reaction time (mean±SD) of each subject to a physical flip in the direction of motion. This reaction time, measured in a separate control experiment, serves as an estimate of when the actual percept switch occurred relative to the moment of the button press. Bottom panels in Figures 5 and 6 show the covariograms averaged (±SE) across all four subjects.

Figure 5 shows the results obtained for large (>1°) shifts. Note that there were increases in saccade (red) and stimulus jump (blue) occurrence approximately 1 s before the button press, and just prior to the estimated moment of the perceptual switch. Except for saccade trials in subject TG, these increases were statistically significant in the time window from approximately −1.5 to −0.6 s for switches towards the preferred eye in all subjects (Figure 5A). For switches towards the non-preferred eye (Figure 5B), however, the increases in shift rate were considerably lower (0.1 versus 0.2 shifts per second, on average). A significant *difference* between the occurrence of large saccades and large stimulus jumps before the button press was observed only for subject JK for percept switches in both directions (BE-tests, p<0.05, not shown).

Both large saccades and large stimulus jumps were typically preceded by a beep which cued the subjects to make a saccade or warned them about an upcoming stimulus jump (Methods). It is possible therefore that the percept transitions synchronized with the beeps rather than with the subsequent image shifts. It appeared, however, that the effect was strongly reduced when we cross-correlated the beep occurrences with percept switches (see Figure S1), indicating that the percept switches tended to synchronize with the image shifts themselves rather than with the preceding beeps.

Figure 6 shows the results obtained for small (<1°) shifts. In the replay condition (blue), the shift rate for individual subjects increased to ∼0.3 s^−1^ above the baseline starting approximately 1.5–1 s before the button press (mean±SD of individual peaks: 0.31±0.06 s^−1^). This increase was statistically significant for switches toward the preferred eye in all subjects (Figure 6A), and reached a peak value that was on average nearly two times larger than the one observed for large image shifts (c.f., Figure 5A; note scaling differences). Due to individual differences in timing, however, this peak is no longer clearly visible when averaged over subjects. For percept switches towards the non-preferred eye, shift rates were also increased significantly in all subjects (Figure 6B) but all peaks were considerably lower. In the saccade condition (red), however, there were no significant increases in shift rate prior to the button press regardless of the switch direction. The resulting differences between the saccade and replay condition were statistically significant (BE-tests, p<0.05) in all subjects except DB. Thus, for small retinal image shifts, there was a remarkable difference as to how these shifts were brought about. When it was a passive shift, brought about by moving the stimulus on the screen, the probability that this shift was followed by a perceptual switch increased, but when it was an active shift, brought about by a saccadic eye movement, the percept tended to remain stable.

Apart from the above-described peaks at ∼1 s before the button press, all subjects also showed significant decreases in saccade and stimulus jump occurrence approximately 0.5 s before the button press (BE-tests, p<0.05). This transient decrease, which was greatest for the small shifts (∼0.2 and 0.1 shifts per second for small versus large shifts, respectively), cannot simply reflect some sort of refractory period, because it also occurred in cases where there was no preceding peak (e.g. small saccades, Figure 6A). Interestingly, this effect had a shorter lead time than the positive effect of stimulus jumps and large saccades on the switching probability (as the observed troughs lie closer to zero than the peaks) but still well within the estimated reaction time (gray bars). No consistent increases or decreases were found more than 1.5 s before or 0.5 s after the button press, neither for large nor for small shifts.

We considered the possibility that the correlations between percept switches and image shifts depended not only on the prior dominance state, but also on the direction of the image shifts. This was tested by splitting the datasets from Figures 5 and 6 into four different direction categories (left, right, up, down). This additional analysis indicated that neither saccade direction nor jump direction had a significant effect (data not shown).

**Figure 7:**
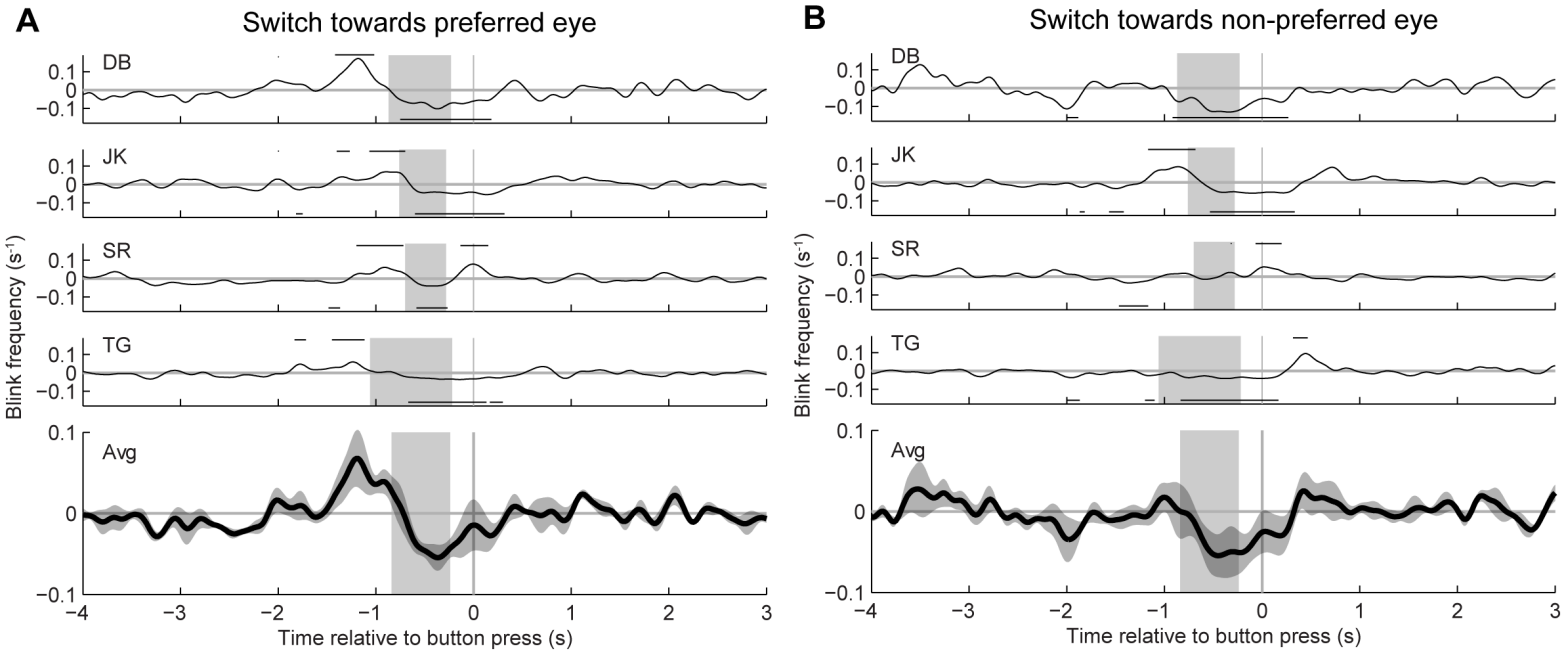
Covariograms of percept switches and blinks. Data are presented in the same way as in Figure 5. A: switches towards the preferred eye. B: switches towards the non-preferred eye. Horizontal lines above and underneath the covariograms indicate significant increases or decreases from baseline, respectively. Note the different scaling of the vertical axis compared with Figure 5 and 6.

Figure 7 shows the cross-correlation analysis for blinks and perceptual switches. Data from saccade and replay trials were pooled in this analysis. The occurrence of blinks increased significantly (BE-test, p<0.05) approximately 1 s before percept switches towards the preferred eye in all subjects. For percept switches towards the non-preferred eye, this increase was statistically significant in only one subject (JK). Just prior to the button press, there was a decrease in blink occurrences for perceptual switches in both directions. This decrease was statistically significant (BE-test, p<0.05) for all four subjects. Note, however, that the influence of blinks was small compared with the effects of saccades and stimulus jumps. For example, the peak and trough values in Figure 7A are, on average, about two times lower than the ones in Figure 5A. We considered the possibility that the observed changes in blink rate resulted indirectly from a synchronization of the blinks with large image shifts or the preceding beeps, but we found no significant temporal correlation between blinks and large shifts or blinks and beeps (see Figure S2; BE-test, p>0.05).

### Relation with onset rivalry

**Figure 8:**
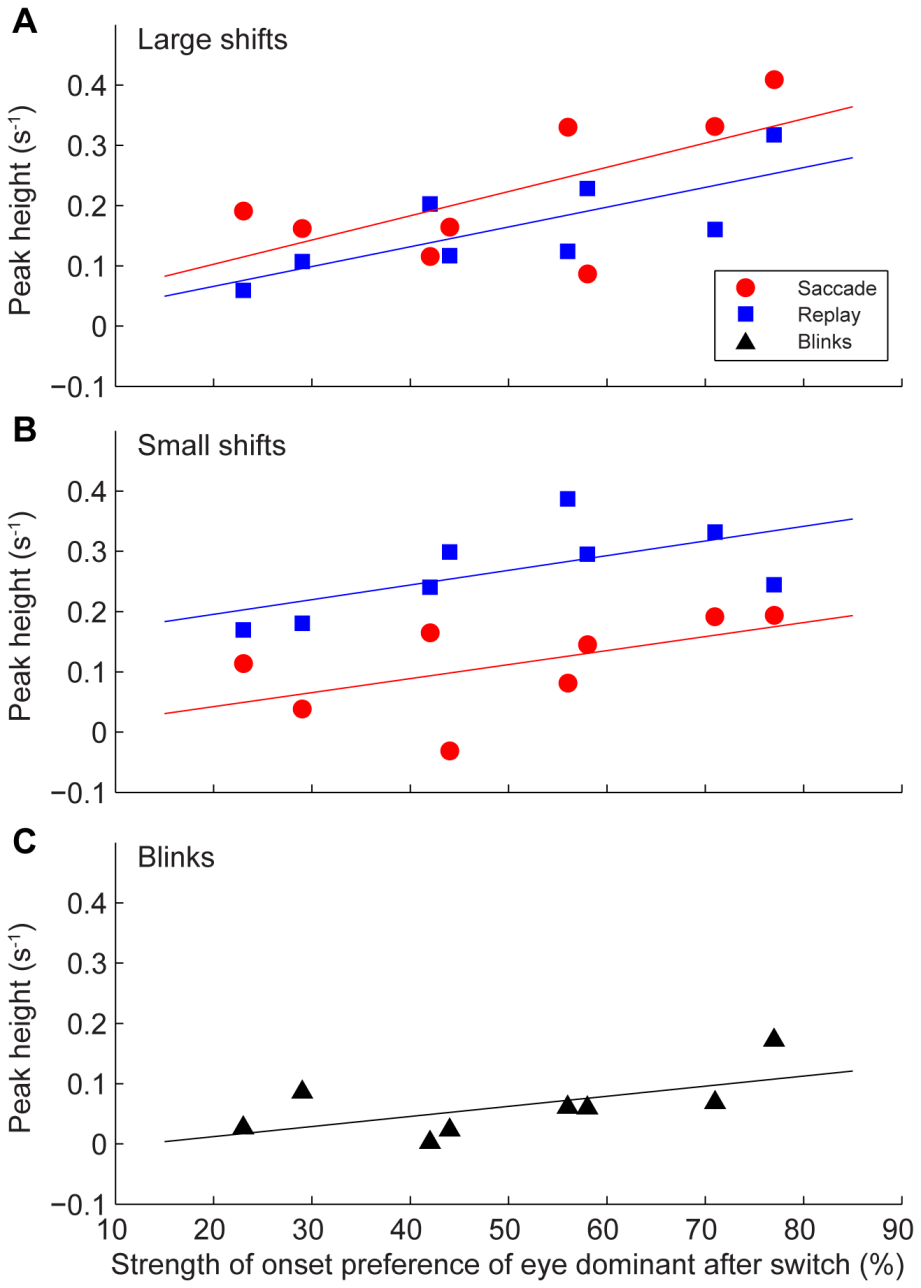
Peak value of the covariogram as function of onset preference strength. A: large retinal image shifts. B: small retinal image shifts. C: blinks. Peaks were determined as the maximal shift rate in the interval from 1.5 to 0.25 s before the button press and plotted against the onset preference of the eye that became dominant after the switch. Linear regression lines show least squares fits to the data.

The observed asymmetry between transitions to the eye which is preferred at stimulus onset and transitions to the other eye suggests that the positive correlation between the occurrence of percept switches and the occurrence of saccades, stimulus jumps and blinks is related to onset rivalry. To explore this possibility further, we examined the strength of these cross-correlations in relation to the strength of the subjects' eye preferences at stimulus onset. This analysis is shown in Figure 8, where we plotted for each subject and each transition the peak value of the covariogram against the probability that the eye to which that percept transition occurred was the dominant one at stimulus onset. This was done for saccades and stimulus jumps >1° (Figure 8A), for saccades and stimulus jumps <1° (Figure 8B), and for blinks (Figure 8C). Note that the strength of the correlations increased systematically with onset preference. This effect was quite strong and remarkably similar for large saccades in the saccade task and large stimulus jumps in the replay condition (Figure 8A). In fact, both the slopes (α) and offsets (β) of two regression lines fitted to these data were not significantly different (mean±SD: α = 0.37±0.11; β = 0.01±0.06). For small saccades and small stimulus jumps, on the other hand, only the slopes of the regression lines were comparable (α = 0.24±0.09). Their offsets differed greatly (β = −0.004±0.05 versus β = 0.15±0.05, respectively), reflecting the fact that the positive correlations between small retinal image shifts and percept switches were much smaller in the saccade condition compared with the replay condition (c.f., Figure 6). For blinks, the increase in peak correlation values with onset preference (Figure 8C) was at the border of significance (t-test, p = 0.05).

A similar analysis was performed on the troughs of the covariograms. This analysis indicated that trough depth was not systematically related to onset rivalry (t-tests, p>0.5; data not shown).

The negative findings for trough depth illustrate that it would be a mistake to think that peak heights of the covariograms should be correlated with the strength of the onset biases simply because there is a reciprocal relation between the onset bias of a subject's preferred and non-preferred eye. Nevertheless, given the basic observation from Figures 6 and 7 that the peak values were on average different for switches toward the preferred and non-preferred eye, one still might suspect that this mean difference alone might fully account for the correlations in Figure 8. It appeared, however, that the adjusted R^2^ values of an alternative model which merely assumed different means (and thus had the same number of parameters as our linear regression model) were lower than the ones obtained for the regression lines shown in Figure 8, which indicates that these regression lines were indeed the better fits to our data. Moreover, for large shifts, the paired difference between peak height for switches towards the preferred and non-preferred eye was significantly correlated with the eye preference across the 4 observers (Pearson's correlation coefficient, r = 0.72; t-test, p<0.05).

### Mean dominance durations

The above analyses demonstrate significant temporal correlations between perceptual switches and retinal images shifts, but not how these image shifts influenced the eye dominance durations. To address the latter question, we compared the mean dominance durations in the saccade and replay condition with the mean dominance durations observed under static viewing conditions.

**Figure 9:**
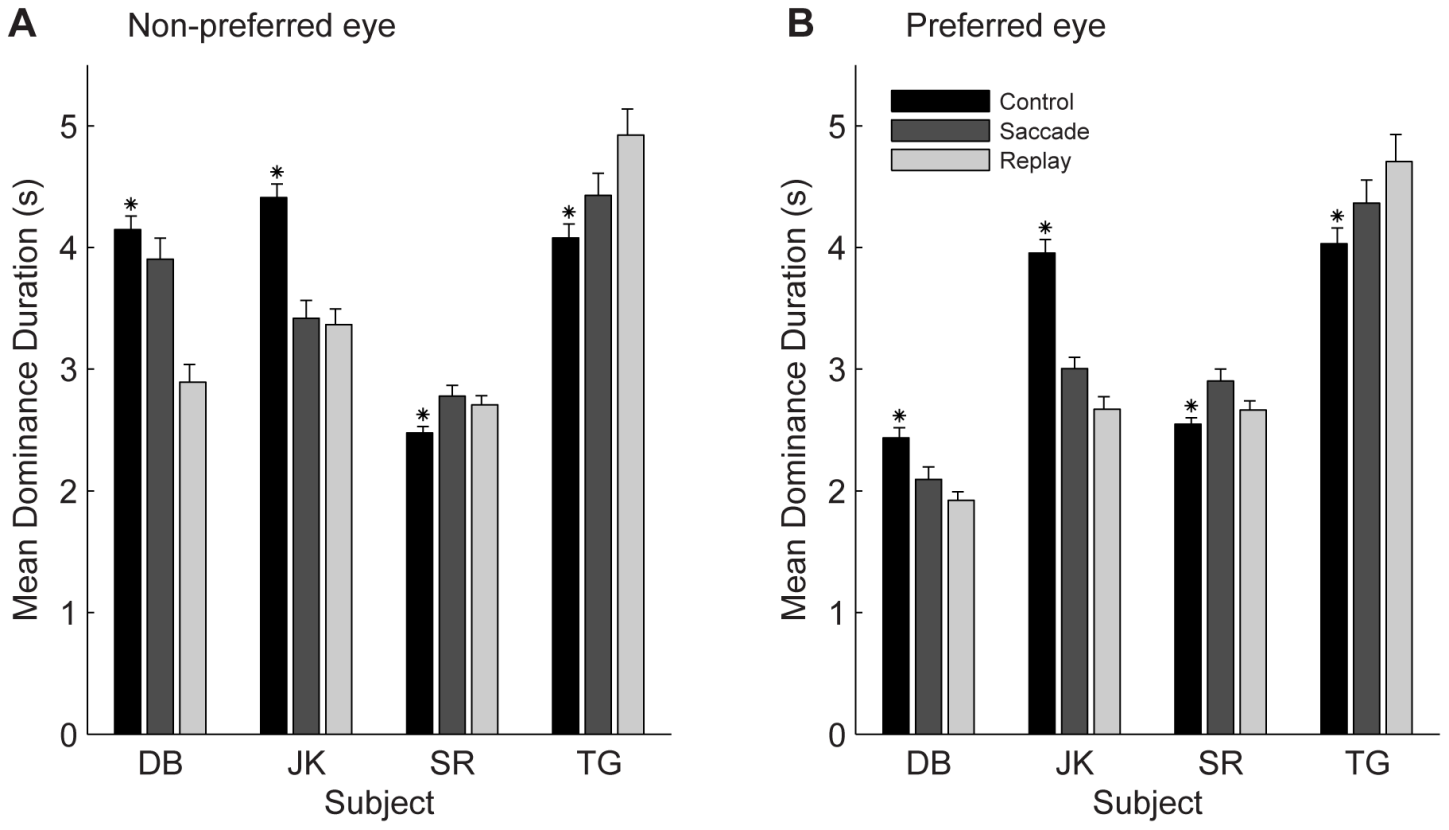
Mean dominance durations. Average dominance durations per subject of the non-preferred (A) and preferred (B) eye under static viewing conditions (black) and in the saccade (dark gray) and replay (light gray) conditions. Error bars indicate ±1 SEM. Asterisks indicate significant differences between control and (pooled) shift conditions (t-test, p<0.05).

The mean dominance durations of both the non-preferred (Figure 9A) and the preferred (Figure 9B) eye percepts were significantly affected by the presence of the image shifts (t-tests, p<0.05, for all subjects), but the effects were mixed across subjects. For both saccade and replay conditions and for both eyes, the mean dominance durations either decreased (subjects DB and JK) or increased (subjects SR and TG) compared with the control condition. While the decreases in mean dominance duration of the non-preferred (left) eye percept in subjects DB and JK may be reconciled with the notion that the image shifts occurring during non-preferred-eye dominance states tend to elicit perceptual switches to the preferred eye dominance, this interpretation does not hold for the decrease in mean dominance durations of their preferred eye percept because the corresponding covariograms (Figures 5B and 6B) did not show similar increases in shift frequency before switches to the non-preferred eye dominance state. This suggest that the changes in rivalry dynamics compared with the static control condition resulted, at least to some extent, from non-specific factors (like task difficulty, perhaps), rather than from the images shifts per se.

Comparing the saccade and replay condition, it is observed that the mean dominance durations of both the left and the right eye percepts tend to be longer after saccades then after stimulus jumps in subjects DB, JK and SR and shorter for subject TG.

### Mean survival time

**Figure 10:**
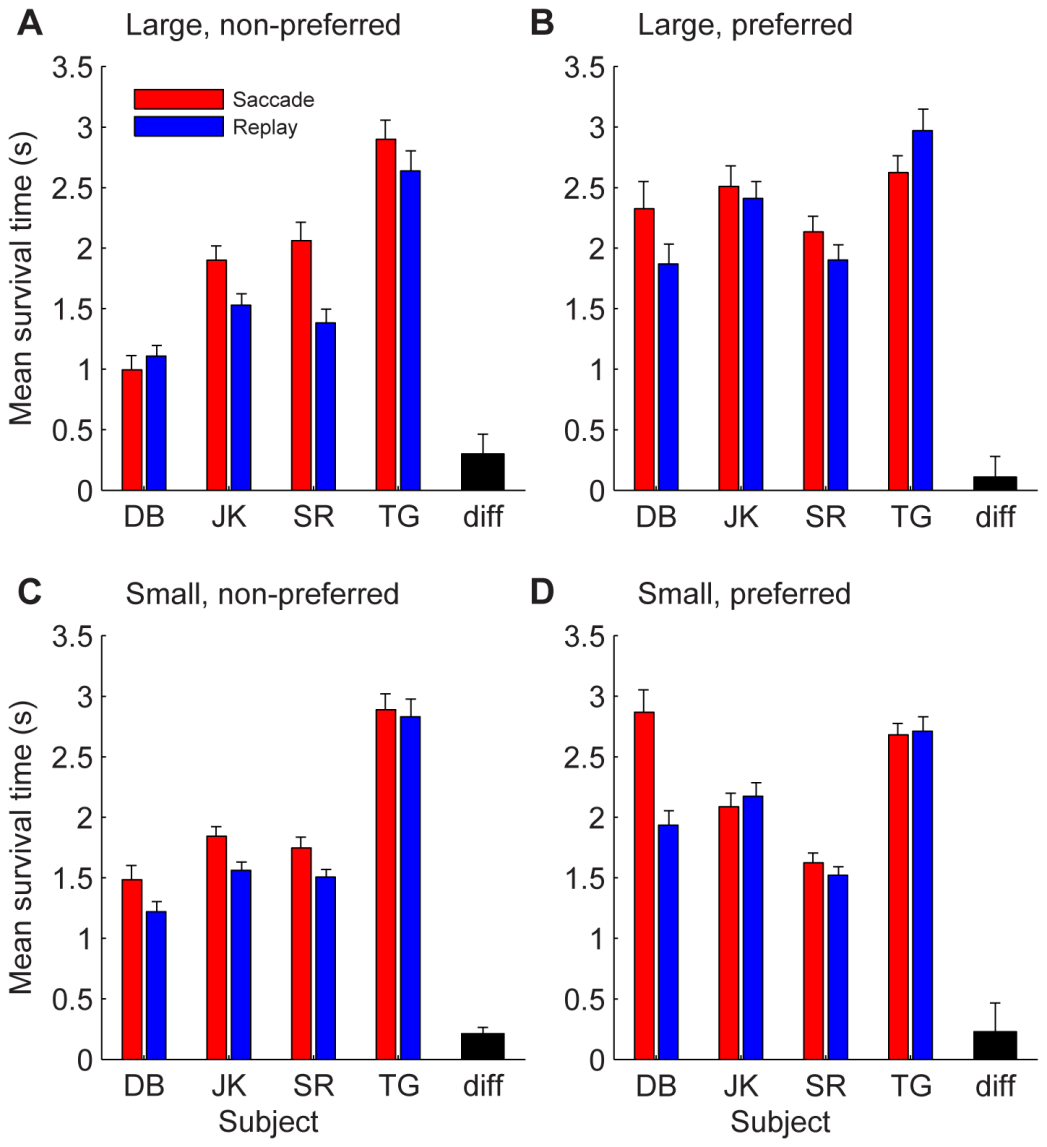
Dominance survival time after retinal image shifts. Average dominance survival time after large (A,B) and small (C,D) retinal image shifts in the saccade (red) and replay (blue) conditions. A,C: Survival of the percept of the non-preferred eye. B,D: Survival of the percept of the preferred eye. Error bars on colored bars show standard deviations. Black bars show the average difference between the two conditions over all four subjects with SEM.

To further address the question how large versus small retinal image shifts affected the durations of dominance states, we also analyzed the mean dominance survival time which quantifies how long the current percept survives after saccades or stimulus jumps (Methods). Figure 10 shows the average dominance survival times of non-preferred (10A,C) and preferred (10B,D) eye percept after large (10A,B) and small-amplitude (10C,D) saccades and stimulus jumps. Note that the average survival times were typically larger in the saccade (red) versus replay (blue) condition. The black bars show the mean difference between the saccades and replay conditions across all four subjects. The observed increases were significantly different from zero for survival times after small shifts in both directions (ANOVA: F = 7.44, p = 0.0065; F = 7.93 p = 0.005 for survival times of the preferred and non-preferred percept, respectively) and after large shifts for the non-preferred eye only (ANOVA: F = 1.79, p = 0.18; F = 4.53, p = 0.0337 for the preferred and the non-preferred percept, respectively).

## Discussion

In the present paper we analyzed the temporal correlations between perceptual alternations and active versus passive retinal image shifts using a new cross-correlation technique, adopted from the field of spike train analysis, which accounts for common input (Methods). Active shifts were produced by saccadic eye movements, while passive shifts were produced by moving the stimulus across the screen in a saccade like fashion. In both experimental conditions, we found significant, positive correlations between retinal image shifts and perceptual switches, but only for switches towards the subjects' preferred eye. For small image shifts (<1°), however, we observed a remarkable dissociation between active versus passive shifts; the probability of small saccades prior to switches showed no significant increase, while small stimulus jumps of the same amplitude and direction showed a robust positive correlation with switches towards the preferred eye. As we will argue below, these findings corroborate our tenet that retinal image shifts trigger onset rivalry, rather than perceptual switches per se, and that this onset rivalry depends at least partly on extra-retinal eye movement signals.

### Onset rivalry

Consistent with previous studies (e.g., [8], [9], [10], [30], [31]) we found significant, positive correlations between retinal image shifts and perceptual switches for both saccades and stimulus jumps. These results are consistent with the notion that the image change resulting from a saccade, rather than the execution of the eye movement per se, is a key factor for switches in awareness [11]. Interestingly, however, we found these positive correlations primarily for switches towards the subjects' preferred eye at stimulus onset (Figures 5 and 6). A similar asymmetry was observed for blinks (Figure 7). These findings are nicely in line with our previous study [19] in which we found that eye dominance after a 4° retinal image shift was biased towards the subjects' preferred eye at stimulus onset. Our present findings thus support the conclusion that retinal image shifts and brief image blanking tend to elicit onset rivalry rather than precept switches per se. This conclusion is corroborated further by our observation that the amplitudes of the cross-correlation peaks increased significantly with increasing strength of the onset preferences of the individual observers (Figure 8).

There are at least two ways in which image shifts could influence rivalry during sustained viewing. First, the transient neural responses associated with a retinal image shift [32] might trigger a reset of the competition process, perhaps because they provide a powerful influx of new information about the stimulus. It could also be that more gradual, fixation-contingent fluctuations in sensitivity influence the ongoing competition. Earlier work on monocular rivalry has shown, for example, that interaction of the stimulus with post-saccadic afterimages leads to changes in perceptual dominance of one grating pattern over the other as well as fluctuations in perceived contrast of a single grating that depend systematically on the nature of the retinal image change produced by a saccade [33]. Thus, saccades can have a profound impact on the perception of static stimuli, depending on their endpoints within the stimulus. Indeed, using orthogonal gratings, van Dam and van Ee [11] found that a saccade only causes a switch in eye dominance if it leads to a retinal image change on the fovea. Note, however, that we circumvented these endpoint contingencies by using dense random-dot motion stimuli; the differences that encouraged binocular rivalry were directional motion signals, not discrepant spatial structures. Hence, even though each saccade and each stimulus jump produced a change in the retinal image, the resulting changes on the fovea were always very similar in nature (i.e., always a random pattern of moving dots) and not important for the competition between the two motion percepts.

We speculate therefore that the observed asymmetries between transitions to the eye that is preferred at onset and transitions to the other eye resulted from visual transients that reinitiate the rivalry process rather than from fixation-contingent (asymmetric) fluctuations in sensitivity. Of course, even in a dynamic motion stimulus, after a retinal image shift, new retinal tissue will be stimulated at the edge of the stimulus. We did not specifically test whether the probability of a perceptual switch depends systematically on the extent to which new retinal tissue is being stimulated, but in a way this is already given to some extent by the difference between large and small shifts. This comparison suggests that size matters, but that it is by no means the only factor that contributes; in the replay condition, peaks in the cross-correlograms had about the same magnitude for small and large stimulus jumps (see Figures 5 and 6).

### Perceptual stability

Interestingly, this asymmetry between switches toward the preferred and the non-preferred eye was observed for small (<1°) stimulus jumps, but not for small spontaneous saccades. This result confirms the notion that small saccades do not interfere with perceptual stability [9] or that saccades are even actively involved in maintaining perceptual continuity [11], [34].

Visual stability during saccades is also observed under natural viewing conditions, when we perceive the world as stable in space despite the retinal image shifts induced by saccades. In contrast, the same eye movements produced by an external cause destroy the stable percept. In agreement with this notion, our subjects reported that watching the stimulus jumping around in the replay condition was very annoying, even though the movement of the stimulus was a copy of their own eye movements made in a previous trial. Visual stability during saccades has also been shown, for example, in oculomotor double-step tasks, in which subjects reach two sequentially flashed targets quite accurately, despite the fact that the retinal information on the location of the second target did not match the eye displacement to reach that target after the first movement [35].

On the other hand, studies using stabilized images by compensation for eye movements [36], [37] or using afterimages [5], [6], [7] have found that dominance durations increase substantially in the absence of eye movements, (although perceptual switches still occur), suggesting that saccades are an important drive for alternations in binocular rivalry. Sabrin and Kertesz [38] found that if the image shown to one eye is stabilized, the predominance of that image gets severely reduced, but that its predominance increases when microsaccades are simulated. This increase, however, was not up to the level of natural viewing. This implies that it is not only the retinal displacement that causes the effect, but also some higher level feature of saccades (e.g. the presence of an efference copy). Our findings that the probability of small saccades is not significantly increased just prior to perceptual switches, whereas the probability of small stimulus jumps is, corroborates the involvement of extra-retinal signals.

### Image stability

Our experiments also demonstrated remarkably robust decreases in the occurrence of saccades, stimulus jumps, and blinks just prior to perceptual switches. These decreases, which were seen in all conditions, are not simply a reaction to previous increases, since they also occurred in conditions in which no increase occurred (e.g. small saccades, Figure 6). Another possible explanation, namely that subjects withhold saccades between the perceptual flip and the button press (as suggested by Van Dam en Van Ee [9], [10]), is also not valid, because significant decreases were also observed in the replay condition, in which the subjects had no influence on the occurrence of the shifts. It thus appears that a short period of retinal image stabilization also increases the probability of a perceptual switch. This notion was further supported by the fact that the covariograms still showed deep troughs even if the percept switches were cross-correlated with all saccades, stimulus jumps and blinks combined (average depth > 0.6 s^−1^; data not shown). These findings are in line with the results of Sabrin and Kertesz [39], who found a decrease in the occurrence of microsaccades over the course of a dominance interval. We therefore speculate that the occurrence of troughs in the covariogram might be related to the so called Troxler effect. It has been found that fixational (micro)saccades counteract visual fading [40], probably by providing new ‘pieces of evidence’ for the present stimulus and thus weakening the amount of adaptation to that stimulus. Fading of the current dominant percept due to image stabilization might contribute to a switch in this way. Indeed, Alais et al. [41] recently published evidence for the influence of adaptation on the rivalry process by showing that the sensitivity for changes in the dominant percept decreases over the course of a dominance state, while it increases for changes in the suppressed percept.

Interestingly, the effect of image stabilization on perceptual switches had a shorter lead time than the positive effect of retinal image shifts and blinking, as the troughs always fell after the peaks in the covariograms (Figures 5–7). Moreover, unlike peak height (Figure 8), trough depth appeared unrelated to the strength of the subjects' individual onset preferences. Both features support the above notion that there are at least two ways in which image shifts could influence rivalry during sustained viewing: one which influences the ongoing rivalry through gradual changes in sensitivity, and another one which reinitiates the competition through strong visually-evoked transients (that are unexpected, unsuppressed or unaccounted for by extra retinal signals).

### Peak timing

Previous studies [10], [31] found peaks in the cross-correlograms at ∼500 ms before the button press. It was suggested that this lead time can be accounted for by delays in the subjects' responses to the perceptual switch because it coincided with the subjects' reaction time to physical stimulus flips [10]. In our experiments, however, the lead time of the peaks was typically larger than subjects' mean reaction time to physical flips in motion direction. However, latencies for unambiguous stimulus flips and perceptual switches induced by rivalrous stimuli need not be the same. Indeed, we recently found that in a motion discrimination task reaction times to rivalrous motion stimuli are consistently increased compared with reaction times to unambiguous stimuli [42]. We thus speculate that the observed timing differences with Van Dam and Van Ee and others [10], [31] are caused by differences between our dynamic versus their static stimuli.

### Dominance durations and survival times

It is tempting to assume that the increased probability of retinal image shifts before perceptual switches observed in the covariograms would lead to a decrease in mean dominance duration and survival times. However, the proportion of switches that is preceded by a shift, even in the condition with the highest peaks, is relatively low (area under the peak, approximately 5–10% on average), leaving many shifts that might possibly delay a switch rather than causing one. Because dominance durations already show quite some variation in the absence of saccades, elongation of part of these intervals would result in a very broad and low peak that is impossible to detect in the covariogram. Predictions on dominance duration from the covariogram are further complicated by the fact that there are not only peaks, but also troughs, meaning that not only shifts, but also the absence of shifts might contribute to the occurrence of a perceptual switch.

Indeed, we found prolonged dominance durations in the shift conditions as compared to the control conditions without saccades or stimulus jumps in two of our subjects and shortened durations in the other two subjects. Overall, both dominance durations and survival times were slightly longer in the saccade conditions than in the replay conditions, supporting the notion that the extra-retinal information that is available when saccades are made, helps to stabilize the percept. However, the differences, although statistically significant, were minimal.

### Blinks

In our experiments blinks occurred more frequently before the button press with which subjects indicated a switch than after it (Figure 7). These findings contrast with earlier studies [9], [31], which reported that blinks occur mainly after the switch. It should be noted, however, that these previous studies have relied on the occurrence of artifacts in their video-based eye movement signals to identify putative blinks while we have recorded movements of the eyelids to measure them directly (Methods). Although in our experiments some subjects did show a significant increase in blink rate after the button press, a much larger increase was seen approximately 1 second before the button press. This suggests that, if anything, blinks tended to elicit switches rather than just synchronize with them.

In this respect it is also interesting to consider the results of studies that have applied intermittent stimulus presentation [18], [20]. In these studies it was found that short (<0.5 s) stimulus interruptions increase the switch probability to such a degree that both stimulus presentations are perceived alternately. If short stimulus interruptions produced by blinking would have had a similar effect, we would have expected a much larger increase in blink occurrence prior to the button press. Even so, our study shows that blinks do occur more frequently before button presses, especially before switches to the subjects' preferred eye (Figures 7 and 8). A parsimonious interpretation of these findings is therefore that blink-induced stimulus interruptions, like saccades, may trigger onset rivalry, rather than (interruption driven) percept switches per se.

## Conclusion

We found a correlation between large (>1°) retinal image shifts and perceptual switches in binocular rivalry. These correlations were stronger for switches towards subjects' preferred eye at stimulus onset, suggesting that, rather than causing percept switches, retinal image shifts trigger onset rivalry. A similar effect was found for blinks.

Small saccades hardly affected binocular rivalry, whereas small stimulus jumps did, indicating that extra-retinal signals associated with saccades (such as efference copy) play a role in the effect of saccades on perceptual switches. This idea is further corroborated by the observation that mean dominance survival times are larger in the saccade condition as compared to the replay condition.

## Supporting Information

Figure S1 **Effects of beeps.** Both large saccades and large stimulus jumps were typically preceded by a beep which cued the subjects to make a saccade or warned them about an upcoming stimulus jump. It is possible therefore that the percept transitions synchronized with the beeps rather than with the subsequent image shifts. Given the variability in saccade (and jump) delay relative to the beep, a stronger effect in the case of beeps would suggest the beeps themselves are important, whereas a weaker effect would suggest it is really the image shift resulting from the saccade (or stimulus jump). To test this, we computed covariograms of button presses with which subjects indicated percept switches, and auditory cues (beeps). A: switches towards the preferred eye. B: switches towards the non-preferred eye. Top panels show the results from individual subjects. Bottom panels plot the mean ± SEM across all four subjects (black lines and gray shaded areas). The vertical gray bar is an estimate (mean±SD) of the moment that the actual percept switch occurred relative to the moment of the button press. The occurrence frequencies of large saccades have been plotted in the bottom panel for comparison (red). The peaks in beep occurrences were lower and the troughs were not as deep as the ones for saccade occurrences, which means that there was a much larger temporal dispersion of the beeps relative to the percept switches. This indicates that percept switches tended to synchronize with the image shifts themselves rather than with the preceding beeps.
(TIF)

Figure S2 **Covariograms of shifts and beeps with blinks.** We considered the possibility that the observed changes in blink rate resulted indirectly from a synchronization of the blinks with large image shifts or the preceding beeps. Therefore we made covariograms of blinks and large retinal image shifts (A) and blinks and beeps (B). Top panels show the results from individual subjects. Bottom panels plot the mean ± SEM across all four subjects. No consistent relation was found between blinks and large shifts, or between blinks and beeps.
(TIF)

## References

[1] Wilson HR (2003) Computational evidence for a rivalry hierarchy in vision. Proceedings of the National Academy of Sciences of the United States of America 100: 14499–14503. 14612564 10.1073/pnas.2333622100 PMC283620

[2] Freeman AW (2005) Multistage model for binocular rivalry. Journal of Neurophysiology 94: 4412–4420. 16148271 10.1152/jn.00557.2005

[3] Noest AJ, van Ee R, Nijs MM, van Wezel RJA (2007) Percept-choice sequences driven by interrupted ambiguous stimuli: a low-level neural model. Journal of Vision 7: 1–14. 10.1167/7.8.10 17685817

[4] Sundareswara R, Schrater PR (2008) Perceptual multistability predicted by search model for Bayesian decisions. J Vis 8: 12 11–19. 10.1167/8.5.12 18842083

[5] Blake RR, Fox R, McIntyre C (1971) Stochastic properties of stabilized-image binocular rivalry alternations. Journal of Experimental Psychology 88: 327–332. 5090924 10.1037/h0030877

[6] Lack LC (1971) The role of accommodation in the control of binocular rivalry Attention, perception, & psychophysics. 10: 38–42.

[7] McDougall W (1903) The Physiological Factors of the Attention-Process (III.). Mind 12: 473–488.

[8] Einhäuser W, Martin KAC, König P (2004) Are switches in perception of the Necker cube related to eye position? The European Journal of Neuroscience 20: 2811–2818. 15548224 10.1111/j.1460-9568.2004.03722.x

[9] van Dam LCJ, van Ee R (2005) The role of (micro)saccades and blinks in perceptual bi-stability from slant rivalry. Vision Research 45: 2417–2435. 15894347 10.1016/j.visres.2005.03.013

[10] van Dam LCJ, van Ee R (2006) The role of saccades in exerting voluntary control in perceptual and binocular rivalry. Vision Research 46: 787–799. 16309727 10.1016/j.visres.2005.10.011

[11] van Dam LCJ, van Ee R (2006) Retinal image shifts, but not eye movements per se, cause alternations in awareness during binocular rivalry. Journal of Vision 6: 1172–1179. 17209727 10.1167/6.11.3

[12] Melcher D (2005) Spatiotopic transfer of visual-form adaptation across saccadic eye movements. Current Biology: CB 15: 1745–1748. 16213821 10.1016/j.cub.2005.08.044

[13] van Boxtel JJ, Alais D, van Ee R (2008) Retinotopic and non-retinotopic stimulus encoding in binocular rivalry and the involvement of feedback. J Vis 8: 17 11–10. 10.1167/8.5.17 18842088

[14] Blake R, Sobel KV, Gilroy LA (2003) Visual motion retards alternations between conflicting perceptual interpretations. Neuron 39: 869–878. 12948452 10.1016/s0896-6273(03)00495-1

[15] Mamassian P, Goutcher R (2005) Temporal dynamics in bistable perception. Journal of Vision 5: 361–375. 15929658 10.1167/5.4.7

[16] Chong SC, Blake R (2006) Exogenous attention and endogenous attention influence initial dominance in binocular rivalry. Vision Research 46: 1794–1803. 16368126 10.1016/j.visres.2005.10.031

[17] Carter O, Cavanagh P (2007) Onset rivalry: brief presentation isolates an early independent phase of perceptual competition. PLoS ONE 2: e343. 17406667 10.1371/journal.pone.0000343 PMC1828625

[18] Klink PC, van Ee R, Nijs MM, Brouwer GJ, Noest AJ, et al (2008) Early interactions between neuronal adaptation and voluntary control determine perceptual choices in bistable vision. Journal of Vision 8: 16.11–18. 10.1167/8.5.16 18842087

[19] Kalisvaart JP, Rampersad SM, Goossens J (2011) Binocular onset rivalry at the time of saccades and stimulus jumps. PLoS One 6: e20017. 21698288 10.1371/journal.pone.0020017 PMC3115950

[20] Leopold DA, Wilke M, Maier A, Logothetis NK (2002) Stable perception of visually ambiguous patterns. Nature Neuroscience 5: 605–609. 11992115 10.1038/nn0602-851

[21] Goossens HHLM, Van Opstal AJ (2010) Differential effects of reflex blinks on saccade perturbations in humans. J Neurophysiol 103: 1685–1695. 20130041 10.1152/jn.00788.2009

[22] Goossens HHLM, Van Opstal AJ (2000) Blink-perturbed saccades in monkey. II. Superior colliculus activity. J Neurophysiol 83: 3430–3452. 10848560 10.1152/jn.2000.83.6.3430

[23] Collewijn H, van der Mark F, Jansen TC (1975) Precise recording of human eye movements. Vision Res 15: 447–450. 1136166 10.1016/0042-6989(75)90098-x

[24] Brainard DH (1997) The Psychophysics Toolbox. Spat Vis 10: 433–436. 9176952

[25] Pelli DG (1997) The VideoToolbox software for visual psychophysics: transforming numbers into movies. Spat Vis 10: 437–442. 9176953

[26] Gerstein GL, Kiang NY (1960) An approach to the quantitative analysis of electrophysiological data from single neurons. Biophys J 1: 15–28. 13704760 10.1016/s0006-3495(60)86872-5 PMC1366309

[27] Aertsen AM, Gerstein GL, Habib MK, Palm G (1989) Dynamics of neuronal firing correlation: modulation of "effective connectivity". J Neurophysiol 61: 900–917. 2723733 10.1152/jn.1989.61.5.900

[28] Perkel DH, Gerstein GL, Moore GP (1967) Neuronal spike trains and stochastic point processes. II. Simultaneous spike trains. Biophys J 7: 419–440. 4292792 10.1016/S0006-3495(67)86597-4 PMC1368069

[29] Ventura Vr, Cai C, Kass RE (2005) Statistical assessment of time-varying dependency between two neurons. Journal of Neurophysiology 94: 2940–2947. 16160097 10.1152/jn.00645.2004

[30] Pheiffer CH, Eure SB, Hamilton CB (1956) Reversible Figures and Eye-movements. The American Journal of Psychology 69: 452–455. 13354811

[31] Ito J, Nikolaev AR, Luman M, Aukes MF, Nakatani C, et al (2003) Perceptual switching, eye movements, and the bus paradox. Perception 32: 681–698. 12892429 10.1068/p5052

[32] Gawne TJ, Martin JM (2002) Responses of primate visual cortical neurons to stimuli presented by flash, saccade, blink, and external darkening. J Neurophysiol 88: 2178–2186. 12424259 10.1152/jn.00151.200

[33] Georgeson M (1984) Eye movements, afterimages and monocular rivalry. Vision research 24: 1311–1319. 6523751 10.1016/0042-6989(84)90186-x

[34] Ross J, Ma-Wyatt A (2004) Saccades actively maintain perceptual continuity. Nature Neuroscience 7: 65–69. 14661023 10.1038/nn1163

[35] Hallett PE, Lightstone AD (1976) Saccadic eye movements to flashed targets. Vision Res 16: 107–114. 1258384 10.1016/0042-6989(76)90084-5

[36] Scotto MA, Oliva GA, Tuccio MT (1990) Eye movements and reversal rates of ambiguous patterns. Perceptual and Motor Skills 70: 1059–1073. 2399079

[37] Pritchard RM (1958) Visual illusions viewed as stabilized retinal images. Quarterly journal of experimental psychology 10: 77–81.

[38] Sabrin H, Kertesz A (1983) The effect of imposed fixational eye movements on binocular rivalry. Perception & psychophysics 34: 155–162. 6634372 10.3758/bf03211341

[39] Sabrin H, Kertesz A (1980) Microsaccadic eye movements and binocular rivalry. Perception & psychophysics 28: 150–154. 7432989 10.3758/bf03204341

[40] Martinez-Conde S, Macknik SL, Troncoso XG, Dyar TA (2006) Microsaccades counteract visual fading during fixation. Neuron 49: 297–305. 16423702 10.1016/j.neuron.2005.11.033

[41] Alais D, Cass J, O'Shea R, Blake R (2010) Visual sensitivity underlying changes in visual consciousness. Current biology: CB 20: 1362–1367. 20598538 10.1016/j.cub.2010.06.015 PMC2918735

[42] Kalisvaart JP, Klaver I, Goossens J (2011) Motion discrimination under uncertainty and ambiguity. J Vis 11: 1–21. 10.1167/11.1.20 21266534

