# Supplementary figures and images for: Influence of Retinal Image Shifts and Extra-Retinal Eye Movement Signals on Binocular Rivalry Alternations
Source: PLoS One. 2013 Apr 12;8(4):e61702. doi: 10.1371/journal.pone.0061702 (PMC3625164; doi:10.1371/journal.pone.0061702)

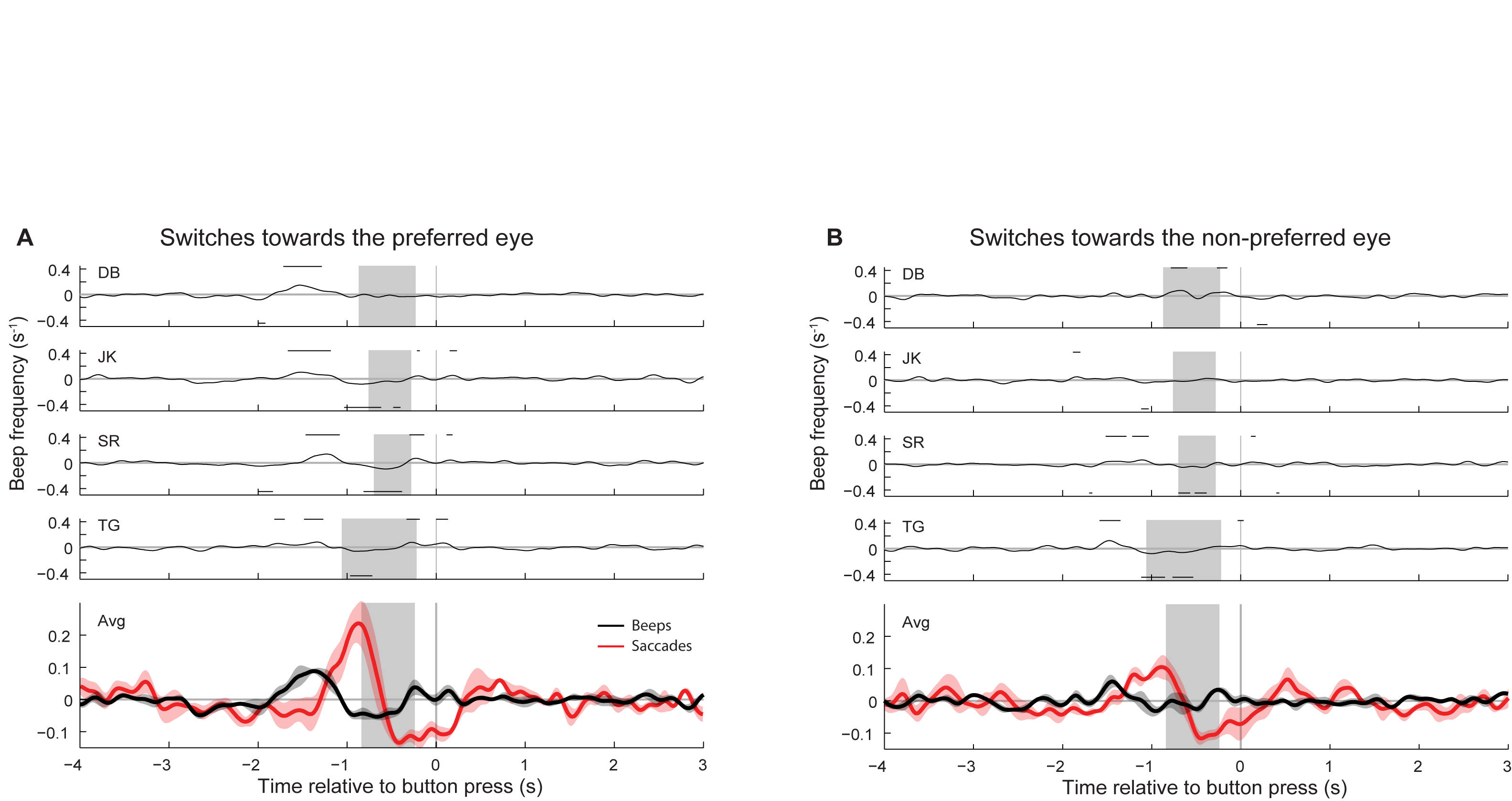

Supplement: Figure S1 — Effects of beeps. Both large saccades and large stimulus jumps were typically preceded by a beep which cued the subjects to make a saccade or warned them about an upcoming stimulus jump. It is possible therefore that the percept transitions synchronized with the beeps rather than with the subsequent image shifts. Given the variability in saccade (and jump) delay relative to the beep, a stronger effect in the case of beeps would suggest the beeps themselves are important, whereas a weaker effect would suggest it is really the image shift resulting from the saccade (or stimulus jump). To test this, we computed covariograms of button presses with which subjects indicated percept switches, and auditory cues (beeps). A: switches towards the preferred eye. B: switches towards the non-preferred eye. Top panels show the results from individual subjects. Bottom panels plot the mean ± SEM across all four subjects (black lines and gray shaded areas). The vertical gray bar is an estimate (mean±SD) of the moment that the actual percept switch occurred relative to the moment of the button press. The occurrence frequencies of large saccades have been plotted in the bottom panel for comparison (red). The peaks in beep occurrences were lower and the troughs were not as deep as the ones for saccade occurrences, which means that there was a much larger temporal dispersion of the beeps relative to the percept switches. This indicates that percept switches tended to synchronize with the image shifts themselves rather than with the preceding beeps. (TIF) [file pone.0061702.s001.tif]

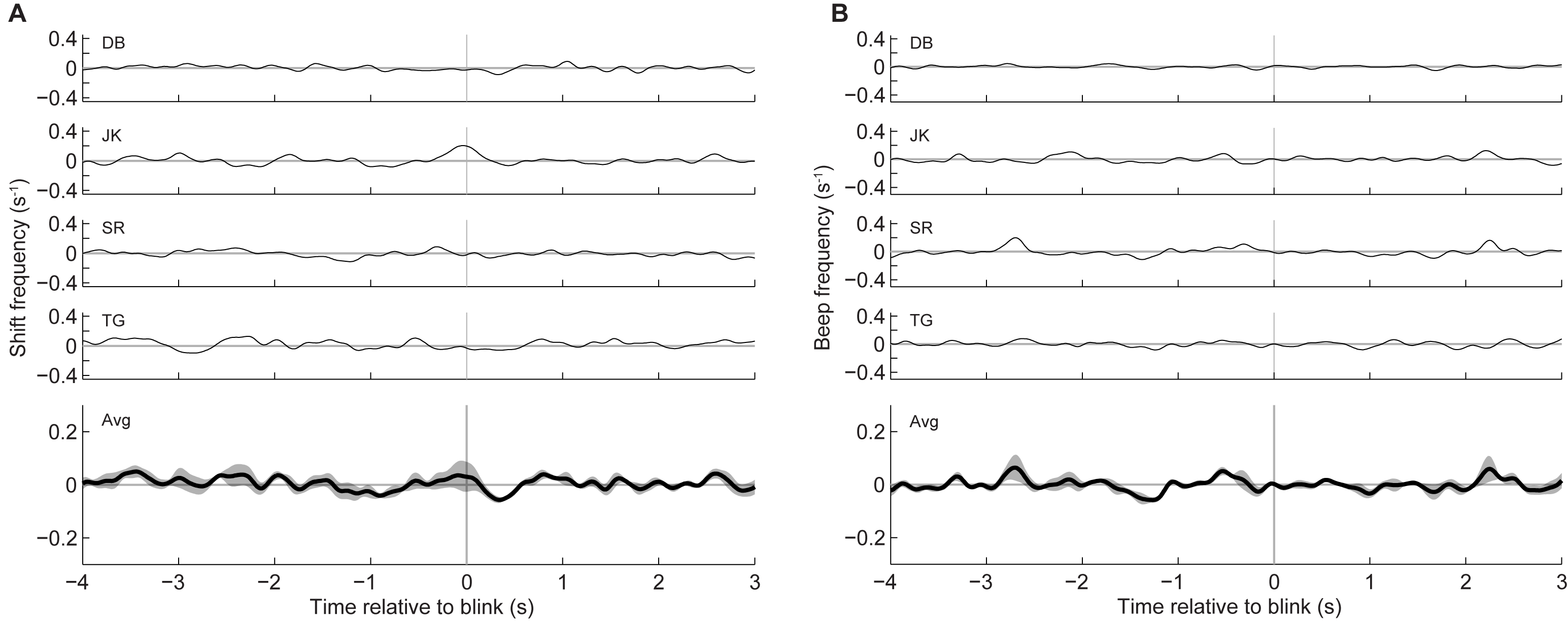

Supplement: Figure S2 — Covariograms of shifts and beeps with blinks. We considered the possibility that the observed changes in blink rate resulted indirectly from a synchronization of the blinks with large image shifts or the preceding beeps. Therefore we made covariograms of blinks and large retinal image shifts (A) and blinks and beeps (B). Top panels show the results from individual subjects. Bottom panels plot the mean ± SEM across all four subjects. No consistent relation was found between blinks and large shifts, or between blinks and beeps. (TIF) [file pone.0061702.s002.tif]
